# The hunger strikes back: an epigenetic memory for autophagy

**DOI:** 10.1038/s41418-023-01159-4

**Published:** 2023-04-08

**Authors:** Patricia González-Rodríguez, Jens Füllgrabe, Bertrand Joseph

**Affiliations:** 1 Division of Biochemistry, Department of Molecular Medicine, Institute of Basic Medical Sciences, University of Oslo, Oslo, Norway; 2 Centre for Cancer Cell Reprogramming, Institute of Clinical Medicine, Faculty of Medicine, University of Oslo, Oslo, Norway; 3 Cambridge Epigenetix Ltd, The Trinity Building, Chesterford Research Park, Cambridge, UK; 4 Institute of Environmental Medicine, Toxicology Unit, Karolinska Institutet, Stockholm, Sweden

**Keywords:** Autophagy, Epigenetics

## Abstract

Historical and demographical human cohorts of populations exposed to famine, as well as animal studies, revealed that exposure to food deprivation is associated to lasting health-related effects for the exposed individuals, as well as transgenerational effects in their offspring that affect their diseases’ risk and overall longevity. Autophagy, an evolutionary conserved catabolic process, serves as cellular response to cope with nutrient starvation, allowing the mobilization of an internal source of stored nutrients and the production of energy. We review the evidence obtained in multiple model organisms that support the idea that autophagy induction, including through dietary regimes based on reduced food intake, is in fact associated to improved health span and extended lifespan. Thereafter, we expose autophagy-induced chromatin remodeling, such as DNA methylation and histone posttranslational modifications that are known heritable epigenetic marks, as a plausible mechanism for transgenerational epigenetic inheritance of hunger.

## Facts

- Human cohorts revealed that fetal, and prepubertal childhood exposure to famine are linked to transgenerational effects on health span and longevity in offspring.
- Animal studies demonstrated that postnatal dietary restriction is associated with extended lifespan for the affected organism and its successive generations.
- Autophagy induction, including upon nutrient deprivation, is associated with improved health span and longevity in multiple model organisms.
- Autophagy induction is associated to epigenetic chromatin changes, including DNA methylation and histone posttranslational modifications.
- Heritable chromatin information, such as DNA methylation and histone posttranslational modifications, contribute to transgenerational epigenetic inheritance.

## Open Questions

- Are histone posttranslational modifications contributing to famine-induced transgenerational epigenetic inheritance in human, as observed in animals?
- Why are prenatal versus postnatal exposure to nutrient deprivation, or autophagy induction, leading to contrasting effects on health span and longevity?
- Most important, are autophagy-induced epigenetic modifications the drivers in nutrient starvation-induced transgenerational effects on offspring?

## A memory of hunger and its impact on offspring longevity

**Fig. 1:**
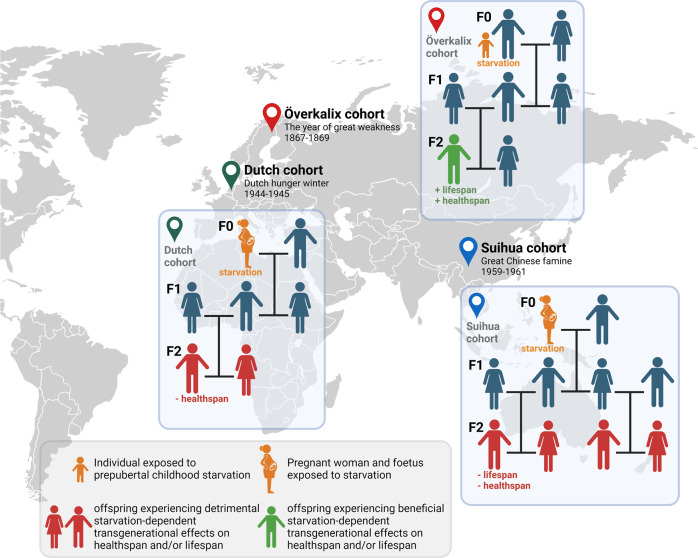
Historical and demographical human cohorts illustrate the impact of famine on human health and longevity, including transgenerational effects up to 2 generations. The Swedish Överkalix cohort (famine 1867–1869), based on individuals exposed to food deprivation during their prepubertal period (5–12 years of age) showed beneficial transgenerational effects in term of health span and lifespan in the grandsons of exposed grandfathers. The Dutch cohort (famine 1944–1945), and the Chinese Suihua cohort (famine 1959–1961) based on individuals, whose pregnant mothers and themselves in utero as foetuses were exposed to nutrients deprivation showed detrimental transgenerational effects in term of health span in the offspring of prenatally exposed fathers (but not mothers) for the Dutch cohort and in term of decreased health and lifespan in the offspring of parents exposed in utero to famine for the Chinese cohort. The image was created with BioRender.com.

In 2010, the TIME Magazine highlighted findings by Lars Olov Bygrov and colleagues describing how hunger can not only affect your own lifespan but also the lifespan of your children and grandchildren [1]. While it is long known that famine can have acute detrimental health-related effects, the use of historical demographical data, the Överkalix cohort, from an isolated parish in the remote northernmost part of Sweden, where a bad harvest (referred as the year of great weakness, “storsvagåret”) led to famine in the mid-nineteenth century (1867–1869) showed that long-term adverse, as well as more unexpectedly favorable transgenerational effects can also be observed (Fig. 1). In their pioneering work, they unveiled a significant connection between the availability, or lack thereof, of food during the prepubertal slow growth period (5–12 years of age) and lifetime expectancy of the second-generation offspring (*i.e*. grandparents to grandchild transgenerational response) [2, 3]. Boys enjoying food availability had grandsons which lived on average 6 years shorter than the grandsons of starved boys. Taking socioeconomic variations into account, the difference in lifetime jumped to 32 years [3]. The increase in longevity of the grandsons was accompanied by a reduction in the risk of cardiovascular disease and diabetes mortality [4, 5]. These results were surprising considering that in clear contrast maternal malnutrition during mid-childhood was linked to increased granddaughters cardiovascular mortality [4–6]. The Uppsala birth cohort multigeneration study (UBCos Multigen), based on much larger Swedish population data set than the Överkalix cohort, confirmed that paternal grandfather’s food access in pre-puberty predicts grandsons’, but not granddaughters all-cause and cancer mortality [7]. Further study performed on the UBCos Multigen cohort established that this male-line transgenerational response to malnutrition is observed for several types of cancers [8]. A study performed in mice showed that paternal malnutrition is associated with an epigenetic and metabolic reprogramming of the mammary tissue of their daughters that in turns higher rates of mammary cancer, however this cancer risk association was not observed in the above human cohort [8, 9]. Hence, in the second-offspring generation, it appears that the mortality rate of men was linked exclusively to their paternal grandfather’s food supply during the prepubertal slow growth period, whereas the mortality rate of women was associated instead to the food supply of their paternal grandmothers, suggesting a sex-specific transgenerational response to starvation during mid-childhood, operating through a paternal line.

Beside children in their prepubertal slow growth period, pregnant women and indirectly their foetuses are also known to represent sensitive groups during a period of famine. Indeed, evidence suggests that the nutritional status during fetal development, reflection of the maternal diet during pregnancy, leads to health outcomes not only on a person as an adult but also on their offspring (reviewed in [10, 11]). An early study on individuals conceived during the Dutch Hunger Winter from 1944 to 1945 suggested that famine exposure in utero was associated with offspring’s poor health in later life with increased chronic disease, however this conclusion was later challenged in a subsequent study performed by the same research team [12, 13]. It remained that adult offspring of prenatally exposed fathers (but not mothers) to famine had higher weights and body mass index than the offspring of prenatally unexposed fathers [13]. Population-based cohort studies based on participants recruited from the Suihua rural area that was affected by the great Chinese famine that occurred between the spring of 1959 and the end of 1961, may offer better understanding of the effect of maternal under-nutrition during gestation. Indeed, these demographical data revealed that prenatal exposure to starvation was associated with elevated risks of developing hyperglycemia, type 2 diabetes, renal dysfunction, and chronic kidney disease in adulthood across two consecutive generations [14–17]. According to the date of birth, F1 subjects were classified as fetal exposed and nonexposed (where the F0 mothers experience famine during their pregnancies). The F2 subjects were classified as having no parents exposed in utero to famine, maternal famine exposure, paternal famine exposure, or parental famine exposure. In the F1 parental generation, prenatal exposure to malnutrition was significantly associated with greater risks of having hyperglycemia and type 2 diabetes [14], as well as of renal dysfunction (as measured by estimated glomerular filtration rate) and chronic kidney disease [15]. One generation down, in the F2 offspring generation, participants from exposed parents, especially those from both exposed parents, still exhibited an increased risk for hyperglycemia and renal dysfunction during adulthood [14–17]. Additional human cohort also based on the great Chinese famine further revealed that parents experience famine in utero or early in life was associated with decreased of their cognitive function. In their offspring, father’s fetal famine exposure was associated with increased depression risk, whereas maternal infant and adolescent famine exposure was associated with decreased cognitive ability [18].

Hence, collectively these human population-based historical cohort studies revealed that in utero fetal, and prepubertal childhood exposure to food deprivation are both linked to long-term health-related effects for the exposed individuals but as well as transgenerational effects in their offspring that affect their diseases’ risk and overall longevity. However, it is important to note the striking difference in the observed transgenerational effects in the offspring based on the time of exposure of the grandparents to nutrient deprivation, with in utero fetal-exposure reported to be linked to detrimental effects whereas in contrast prepubertal childhood exposure was associated to beneficial effects. Collectively, these discoveries infer the existence of “a heritable memory of starvation/hunger”. Nevertheless, by their nature, the human cohorts present clear limitations when it comes to the investigation and possible understanding of the molecular and cellular mechanisms behind nutrient-starvation-induced beneficial effects on longevity. In addition, it could also be argued that an obvious caveat is that some of the observed effects in human populations that exhibit large degree of variation, might be purely due to survival of the fittest, hence starvation could remove the “weak” ones and led to stronger offspring.

More than a century ago, stunted female rats from poor nutrition were reported to live longer, as compared to control stock rat [19]. Later studies in rats showed that a reduction in maternal food intake during pregnancy produced female offspring with shorter lifespan [20], hence replicating some of the observation made with the human cohorts. Since, multiple organism models, ranging from yeast (*Saccharomyces cerevisiae*), fruit flies and sawflies (*Drosophila melanogaster* and *Athalia rosae*), nematodes (*Caenorhabditis elegans*), rodent (*Mus musculus*, and *Rattus norvegicus*), as well as non-human primate such as rhesus monkeys (*Macaca mulatta*) and gray mouse lemurs (*Microcebus murinus*) have been developed that reproduce the observation that a postnatal dietary restriction history is associated with an extended lifespan for the organism itself and for some of these models even its offspring [21–26]. However, the beneficial effects of nutrient restriction on longevity is not universal, as its effects vary between mouse strains (*i.e*. C57BL/6, DBA/2, and B6D2F1) and it is not observed in houseflies (*Musca domestica*) [27, 28].

## Role for autophagy, cellular response to starvation, in lifespan expansion

An essential ability of single cells and by extension of multicellular organisms is to sense nutrient fluctuations in their environment and to adjust their consumption and response accordingly. This adaptation enables cells to survive during periods of nutrient deficiency, and to grow and proliferate when nutrients are plentiful. The capability of cells to sense and respond to nutrient availability and lack thereof in their environment is a prerequisite for life. In fact, nutrient shortage is a selective pressure that has shaped the evolution of most cellular processes [29].

**Fig. 2:**
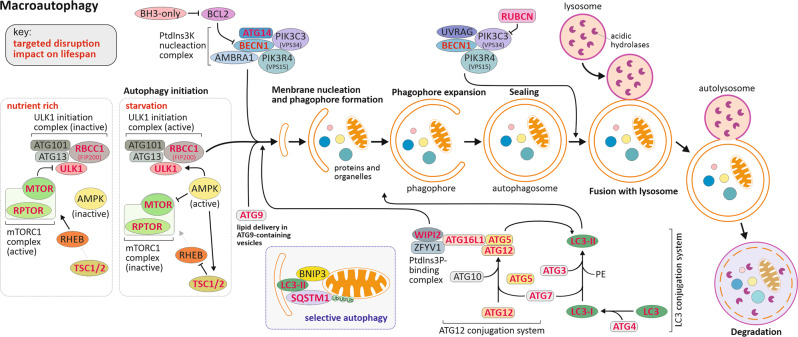
The autophagy core machinery with indication of which components manipulation have been shown to impact on longevity in various organisms. Bulk autophagy starts with the stepwise engulfment of cytoplasmic material by the phagophore, which matures into a double-layered vesicle named an autophagosome. AMP-activated protein kinase (AMPK/PRKAA2) and MTOR-containing mTORC1 complex promote and repress autophagy induction, respectively, through phosphorylation of ULK1 (unc-51 like autophagy activating kinase-1) at distinct residues. Under nutrient rich conditions, AMPK is inactive but mTORC1 is active and phosphorylates and inactivates ULK1. When nutrient starvation occurs, AMPK is activated and mTORC1 is inhibited by AMPK through the phosphorylation of TSC1/2 (TSC complex subunit 1/2) and RPTOR/RAPTOR (Regulator-associated protein of MTOR). Subsequently, ULK1 can interact with and be activated by AMPK-mediated phosphorylation, thereby initiating autophagy. Activation of the ULK1-containing initiation complex triggers phagophore formation by phosphorylating components of the BECN1 (Beclin-1) and ATG14-containing class III phosphatidylinositol 3-kinase (Ptdlns3K) nucleation complex. The activated Ptdlns3K nucleation complex generates PtdIns3P, which leads to the recruitment of the effector proteins WIPI2 (WD repeat domain phosphoinositide-interacting protein 2) and ZFYV1 (Zinc finger FYVE domain-containing protein 1). The expansion of the phagophore requires two ubiquitin-like conjugation systems. The ATG12-conjugation system that supports in the formation of ATG12–ATG5-ATG16L1 ternary complex, which in turn promotes the second conjugation reaction. The second system, the LC3 conjugation system, involves the conjugation of phosphatidylethanolamine (PE) to MAP1LC3/LC3 (microtubule associated protein 1 light chain 3, in mammals). Lipid conjugation converts the soluble form of LC3-I into a phagophore membrane-bound LC3-II form that functions in phagophore expansion, and in cargo recognition of ubiquitinated proteins and organelles, including upon selective autophagy with the involvement of autophagy receptors, *e.g*. BNIP3 (BCL2/adenovirus E1B 19 kDa protein-interacting protein 3), and Ub-dependent autophagy receptors, *e.g*. SQSTM1/p62 (Sequestosome-1). As a result of membrane expansion and sealing, the autophagic cargo becomes sequestered within the autophagosome. Autophagy completion involves the fusion of the mature autophagosome with a vacuole or lysosome. An alternative Ptdlns3K complex containing UVRAG (UV radiation resistance-associated gene protein), negatively regulated by RUBCN/RUBICON (Run domain Beclin-1-interacting cysteine-rich domain-containing protein), has been reported to regulate the processes of fusion between autophagosomes and lysosomes. Docking and fusion of the outer autophagosomal membrane with that of the lysosome exposes the inner vesicle to the lysosomal lumen, where acidic hydrolases degrade and recycle the macromolecular components for cellular use. Key: Component of the autophagic machinery whose alterations are reported to impact on lifespan are highlighted with red bold text.

Cells have developed an evolutionary conserved catabolic process, termed macroautophagy (hereafter referred as autophagy), to cope with stress conditions such as nutrient starvation [30–32]. During starvation-induced autophagy, non-essential cellular components are packed into double membrane vesicles, known as autophagosomes, to be thereafter broken down when these autophagosomes fuse with lysosomes, which are rich in proteolytic enzymes; the resulting metabolites can then be reused for core biosynthetic processes or energy production. Proteins constitute a reservoir of amino acids that can be mobilized upon autophagy induction to be recycled and used to sustain new protein synthesis required under starvation conditions. In addition, under those periods of starvation, amino acids are catabolized for the production of energy required to fuel the particular needs of certain vital organs. Hence, autophagy serves as an internal source of stored nutrients under conditions of nutrient limitation. The autophagic catabolic process is also involved in the sequestration and dismiss of protein aggregates, damaged organelles, or pathogenic organisms. Under physiological conditions, a basal level of autophagy is required to maintain cellular homeostasis [33]. Under stress conditions, initiated by a range of extra- or intracellular stress stimuli, autophagy can be enhanced to protect the organisms [34]. Consequently, disturbances in this biological process have been linked to many human diseases, including neurodegenerative diseases, metabolic disorders, psychiatric disorders, cardiovascular diseases, and cancers [35]. Thus, autophagy is divided into mechanistically distinct steps, including induction, cargo recognition and selection, autophagosome formation and fusion with the lysosome and breakdown of the cargo. At the molecular level, the membrane-rearrangements and cargo recognition are tightly regulated and mediated by a core set of Autophagy-related (ATG) proteins [35]. The autophagic core machinery is out of the scope of the present article, hence for details we refer to reviews on the topic, as well as the illustration in Fig. 2 [36, 37].

At the molecular level, an evolutionary conserved serine/threonine protein kinase, the mechanistic target of rapamycin (MTOR), when part of mTOR complex 1 (MTORC1), functions as a primary nutrient sensor, in particular for amino acids, linking the activation *versus* repression of the cellular processes to the nutritional supply [29]. When nutrients are abundant, active MTORC1 promotes numerous anabolic processes, such as protein, nucleotide and lipid biosynthesis, while catabolic processes, including autophagy, are repressed. Upon amino acids limitation, MTORC1 is rapidly inactivated, and autophagy induced, allowing the cells to cope with the occurring nutrients starvation (Fig. 2) [38]. Hence, MTOR has emerged as a nutrient sensor of a plethora of extracellular and intracellular cues, and one of the central regulators of autophagy induction. Nutrient-sensing pathways, such as insulin/insulin-like growth factor 1 signaling and mTOR inhibition are thought to act as a determinant of increasing longevity in fruit flies [39, 40]. In *C. elegans*, inhibition of TOR, known to lead to autophagy induction, is associated to a strikingly doubling of lifespan for the nematode [41]. Likewise, in *D. melanogaster* the modulation of TOR, by the overexpression of TSC1 and TSC2 (tuberous sclerosis complex genes 1 and 2) that act together to inhibit TOR, or by the expression of dominant-negative forms of TOR all cause lifespan extension of the fruit flies (Fig. 2) [40].

Supporting evidence suggests that autophagy declines during aging, which might contribute to increased cellular stress and accumulation of damaged organelles that could lead to the development of age-related diseases and reduced lifespan expectancy [42]. In that regard, centenarians are excellent subjects to uncover potential mechanisms involved in a healthy aging and consequently human longevity. Remarkably, the genome wide analysis of the transcriptome by RNA sequencing of centenarians, centenarian-children, as well as their spouses revealed that, among the differentially expressed genes, the autophagy pathway was found to be significantly maintained by Centenarians. Remarkably the overexpression of one of these centenarians differentially expressed autophagy-related genes, namely WIPI1 (Atg18a in the fly) in *D. melanogaster* was shown to extend their lifespan [43]. The analysis of the expression levels of 40 mTOR pathway genes in a unique cohort of Dutch families with extended survival across generations, showed that RPTOR (Raptor) gene was found to be expressed at a lower level in the long-lived individuals, a differential expression that was conserved in their offspring, suggesting an association with familial longevity [44]. The expression of RUBCN/Rubicon (run domain Beclin-1-interacting and cysteine-rich domain-containing protein), a negative regulator of autophagy, increases in aged worm, fly and mouse, suggesting that an age-dependent increase in RUBCN could reduce autophagy activity over time [45]. Knockdown of *RUBCN* extends worm and fly lifespan and ameliorates several age-associated phenotypes. RUBCN was also found to be suppressed in several long-lived worms and calorie restricted mice [46]. In addition, studies report that the expression levels of a plethora of core component for the autophagic machinery, such as ULK1, ATG7, MAP1LC3, LAMP2, SQSTM1/p62, or BECN1 are found to be significantly affected in aged organisms [47–49]. Moreover, there is evidence that artificial maintenance of the basal autophagy activity level upon aging, for example via the overexpression of ATG5 in mice, or Atg8a, Atg1 or SQSTM1 in fruit flies or SQST-1 in nematodes increase lifespan [50–53]. A knock-in gain-of-function point mutation in *Becn1* which disrupt BECN1-BCL2 interaction and constitutively activates autophagy has also been shown to extend lifespan in mice [38].

In contrast, the targeted disruption of core autophagy-related genes (e.g. *ULK1*, *ATG5*, *ATG7, ATG9, BECN1*, or their homologs) in yeast, flies, worms and mice results in a block of starvation-induced autophagy which is linked to reduced viability [54–58]. It appears that prolonged lifespan upon starvation, as well as other autophagy stimuli, is only observed in autophagy-proficient organisms. *D. melanogaster* lacking the core autophagy regulator Atg7, that is required for starvation-induced autophagy in the flies, are viable but exhibit reduced longevity. These flies are hypersensitive to nutrient and oxidative stress and accumulate ubiquitin-positive aggregates in degenerating neurons [56]. In *C. elegans*, atg7 is required for longevity in the dietary restriction eat-2 mutant, but not in the insulin-resistant daf-2 longevity model [58, 59] Depletion of another core autophagy-related gene, Beclin-1 (bec-1) in *C. elegans*, abolished the beneficial effects on longevity of resveratrol or calories restriction [57]. Taken together, these results suggest that suppression of autophagic activity is one of the signatures of aging and that counteracting this decrease can contribute to longevity in multiple organisms.

The direct effect of autophagy modulation on one individual’s cancer onset risk remains controversial (reviewed in [60, 61]). As an illustration, taking advantage of short-hairpin RNA targeting *Atg5* expression placed under the control of a doxycycline-dependent promoter allowing the conditional and inducible silencing of this core autophagy gene in mouse, it was shown that the systemic autophagy inhibition from the age of 2 months accelerated aging and aged-associated pathologies, hence reduced lifespan. In contrast, the transient inhibition of autophagy, achieved by the restoration of *Atg5* expression at the age of 4 months age in the mice, provided a near complete recovery from the aging phenotype and extended lifespan. However, autophagy-restored mice still succumb earlier than the control mice due to an increase in spontaneous tumor formation [62].

The popular dietary regimes based on reduced food intake, i.e. exposing ourselves voluntarily to hunger, thought to promote longevity, such as calorie restriction, or intermittent time-restricted feeding, also known as intermittent fasting, are all prominent inducers of autophagy leading to the assumption that autophagy is the key pathway in the induction of longevity. Calorie restriction, referred to as a reduction in dietary calorie intake without malnutrition, has been demonstrated to be the most physiological inducer of autophagy to extend longevity across animal models, Epidemiologically, calorie restriction in humans moderates intrinsic processes of aging through cellular and metabolic adaptations and decrease the risk factors in age-related diseases in humans. Therefore, autophagy is an integral mediator of calorie-restriction-induced lifespan extension with the potential to be inherited since the beneficial effect of this dietary regime on longevity fail if autophagy is inactivated.

As an illustration, the inactivation of two essential autophagy genes, *bec-1* and *Ce-atg7*, which are orthologs of human *BECN1* (Beclin-1) and *ATG7*, respectively, barred the longevity phenotype of the *C. elegans* dietary restriction mutant (eat-2(ad1113) animals [59]. Likewise, the silencing of SIRT1 (Situin-1) expression in the nematode, which is required for autophagy induction upon dietary restriction, abrogated the lifespan extension in the worm [57]. The effect of calorie restriction has been also studied in non-human primates, but the results are conflicting. A 20-year longitudinal adult-onset calorie-restriction study in rhesus monkeys reported a positive impact of this diet on health, age-related survival, and all-cause survival; hence an overall increased longevity [63]. However, another longitudinal study also conducted on rhesus monkeys failed to report an effect of calorie restriction on longevity [64]. Reason for this discrepancy has been suggested to rely in the source of the monkeys, the feeding practices and diet composition, as well as the age of onset of calorie restriction [24]. The established links between calorie restriction, autophagy, longevity and healthy aging has brought interest of the public and scientific communities into caloric-restriction mimetics (*e.g*. aspirin, resveratrol, spermidine) that could activate autophagy, and prolong life- and health span without the need to cut calories [65]. Intermittent fasting that restricts food intake to specific hours of the day, has also gained interest as a potential anti-ageing treatment [66]. In fruit flies, such dietary regime has been shown to significantly extend the health span and lifespan (by up to 25%) [67, 68]. The beneficial effects of this dietary regime rely on a circadian rhythm-dependent activation of autophagy [68]. In fact, autophagy has long been known to be regulated by the circadian clock such that it peaks at night [69, 70]. Considering that both circadian regulation and autophagy are highly conserved processes, this study provides the interesting possibility that behavioral or pharmaceutical interventions that promote circadian-regulated autophagy could be used to promote healthy aging and increase longevity even in human.

Inhibition of MTOR/TOR kinase by its name defining inhibitory drug, rapamycin (also known as Sirolimus, isolated from *Streptomyces hygroscopicus*, and certainly one of the best-characterized pharmacological inducers of autophagy) or its analogs (rapalogs), lead to an extension of lifespan in several organisms, seemingly mimicking the effect of dietary restriction. The effects of both chronic treatment and brief pulse of the drug have been considered. Feeding rapamycin to adult *Drosophila* or adult *C. elegans* reproduces the lifespan extension observed with some TOR-related mutants (*4E-BP* (Eukaryotic translation initiation factor 4E-binding protein) null mutant flies, *daf-15* (abnormal Dauer Formation-15 and RPTOR homolog) and *rheb-1* (RHEB (Ras homolog enriched in brain) homolog) deficient worms) [71, 72]. In mice, chronic exposure to rapamycin using dietary encapsulated rapamycin extends lifespan [73–75]. Additionally, rapamycin administration in food, starting at ~9 months of age, increased the lifespan of females by 18% and male by 10% [74]. Rapamycin even fed late in life, starting at ~20 months of age, was able to increase the lifespan of females by 15% and male by 9% [73]. In mice, rapamycin treatment can delay several age-related diseases, such as cognitive decline, cardiac dysfunction, and cancer [75–77]. However, even at low doses, long-term rapamycin administration can cause adverse effects, and therefor transient administration of the drugs has been considered. In female *D. melanogaster*, a brief, early rapamycin treatment of adults extended lifespan to the same degree as lifelong dosing [78]. Transient rapamycin treatment, for a 3 months period, is sufficient to increase life expectancy by up to 60% and improve measures of health span in middle-aged mice [78, 79]. The long-term effects on longevity of a transient induction of autophagy by rapamycin, and potentially other autophagy inducers, once more supporting the existence of a memory of this biological process.

Additional natural compounds which are inducers of autophagy, and considered to be caloric-restriction mimetics, have been reported to promote longevity. For example, spermidine a natural polyamide, originally isolated from semen, but also found to be enriched in wheat germ, grapefruit and soybean, induces autophagy and promote longevity in autophagy-proficient model systems [80–82]. Lifelong access to drinking water supplemented with spermidine prolonged the median lifespan of female mice by ~10% [81]. Another example, resveratrol, a polyphenol found in red wine, and a potent activator of Sirtuin-1 (SIRT1), induces SIRT1-dependent autophagy and improve longevity in several organisms. Early study, revealed in the budding yeast *S. cerevisiae*, that resveratrol treatment stimulating the SIRT1 ortholog Sir2, was able to significantly extend lifespan by a striking ~70% [83]. These observations of increased lifespan and Sir2/SIRT1 dependency were further extended to the metazoans *C. elegans* and *D. melanogaster* [84]. Thereafter, autophagy was shown to mediate the lifespan expansion of *C. elegans* by resveratrol, as this compound, as well as other Sir2/SIRT1 activators were only able to prolong the lifespan of autophagy-proficient nematodes [57]. However, multi-centers comparative studies failed to show that resveratrol treatment can impact on the lifespan of genetically heterogeneous male and female mice [74, 85], suggesting that the longevity promoting effect of resveratrol may not be conserved in higher organisms. Even the widely administrated drug aspirin, also known as acetylsalicylic acid, a nonsteroidal anti-inflammatory drug used to reduce pain, fever, and inflammation, can recapitulate features of caloric restriction, and stimulate the induction of autophagy by virtue of its ability to inhibit the acetyltransferase activity of EP300/p300 [86, 87]. The induction of autophagy by aspirin, or its active metabolite salicylate is observed in both mice and nematodes, but not in the EP300 ortholog *cpb1*-deficient nematodes [87]. it should be noted that whether treatment with aspirin, beyond its health promoting effects, acting as a caloric-restriction mimetic can promote longevity, remains to be formally established. However, a multi-centers study showed that aspirin led to increase lifespan of genetically heterogeneous male (but not female) mice [88]. In addition, the Finnish centenarian study, a population-based survey of subjects over 100 years old, suggests that subjects taking aspirin had a statistically significant increase in survival as compared to subjects who did not take the drug [89]. As illustrated in Table 1, beyond the above-mentioned examples, the list of natural and synthetic compounds activating autophagy and increasing longevity in various model organisms is increasing by the day.

**Table 1:** List of compounds that regulate both autophagy and longevity.

Pharmacological agent/Compounds	Mechanism of action/Impact on autophagy	Effect on health and lifespan	References
Acetylsalicylic acid and derivatives (C8-SA)	Anti-inflammation effect, autophagy induction by inhibition of EP300/p300; Activation of mitochondria unfolded protein response	Increase lifespan of genetically heterogeneous male mice; Increase lifespan in worms	[86, 87]
Alpha-ketoglutarate (AKG)	Reduce mTOR activation by inhibiting ATP synthase	Prolong lifespan in worms and flies, but reduces reproduction	[144]
Cannabidiol	SIRT1-dependent autophagy induction	Prolong lifespan and increased neuronal health in worms	[145]
Flavonoid 4,4′-dimethoxychalcone (DMC)	Induces autophagy in an mTOR-independent manner, but depends on GATA transcription factors	Increase lifespan of yeast, worm and flies	[146]
*Ganoderma lucidum*, dietary supplements	Induce autophagy in an mTOR-dependent manner and stress resistance by reducing the levels of Fibrilarin 1(FIB-1) and LGG-1	Increase longevity in worms and human cells	[147]
Glucosamine (GlcN)	Promotes autophagy via an mTOR-independent manner	Extend lifespan in nematodes	[148, 149]
Nordihydroguaiaretic acid (NDGA)	Inhibition of EP300/p300 and activation of autophagy	Increase lifespan in flies and mice	[150]
Pyrroloquinoline quinone (PQQ)	Activation of autophagy via Insulin/IGF1 signaling pathway	Extends lifespan in worms by 31%	[151]
Metformin	Promote autophagy via AMPK	Extends lifespan in nematodes and mice	[152]
Nicotinamide riboside (NR) and nicotinamide mononucleotide (NMN), dietary supplements	Increase autophagy and mitophagy	Increase lifespan in worms, flies and mice	[153]
Rapamycin	Autophagy induction by direct inhibition of mTOR	Extends lifespan in worms, flies and mice	[154]
Resveratrol	SIRT1-dependent autophagy induction	Improve longevity in yeast, worms, flies and mice	[155]
SGLT2 Inhibitors	Mimics calorie restriction by inducing glycosuria. Promotes upregulation of AMPK, SIRT1 and mTOR inhibition	Appear to be a promising treatment extending longevity and reduce oxidative stress	[156]
Shatavarin IV	Via eat-2 activation promotes autophagy by promoting the expression of autophagy-related genes expression in an mTOR-dependent manner	On dietary restriction prolonged lifespan	[157]
Spermidine	Autophagy induction, reduce inflammation, lipid metabolism, regulation of cell growth, proliferation and cell death	Prolong median lifespan of female mice	[158]
Verapamil	Inhibition of calcineurin activity and activation of autophagy	Prolong lifespan, improve health span and delay senescence in worms	[159]

Does autophagy induction, including upon nutrient starvation, always promotes longevity? This is certainly a matter for debates. Indeed, as mentioned earlier on treatments with autophagy inducers, often caloric-restriction mimetics, do not necessarily lead to increased lifespan across species. For example, whereas rapamycin/rapalogs appear to promote longevity in all the tested model organisms, resveratrol was unable to increase lifespan in mice [74]. The observed effect may also be sex-dependent, as illustrated by the beneficial effect of aspirin on male but not female mice lifespan [88]. Finally, even the prevailing notion that autophagy is beneficial for longevity has even been challenged. Indeed, a study presented data indicating that autophagy induction coupled with increased mitochondrial permeability is in fact detrimental to the lifespan of nematode [90, 91]. Elevated autophagy unexpectedly shortens the lifespan of *C. elegans* lacking *sgk-1* (serum/glucocorticoid regulated kinase-1, and homolog of SGK) or *rict-1* (rapamycin-insensitive companion of TOR, and homolog of RICTOR), two negative regulators of autophagy, because of a concomitant increase in mitochondrial permeability in these mutants [91, 92]. Likewise, overexpression of *vdac-1* (homolog of voltage-dependent anion channel VDAC1), increase mitochondria permeability by regulating the opening of the mitochondrial permeability transition pore in promoted autophagy but decreased lifespan in *C. elegans*. Inhibition of autophagy, targeting *bec-1* or *lgg-1* expression, or even silencing *vdac-1*, restored the normal lifespan of *sgk-1* mutant [91]. However, it should be highlighted that it remains unclear from these studies whether the decrease in lifespan observed in the nematode manipulating genes that affect both autophagy and mitochondrial functions is the result of a decreased longevity or increased toxicity. In fact, mice lacking liver *Sgk1* gene expression are more sensitive to liver ischemia/reperfusion injury and thus would live shorter as a result.

## The missing link: an epigenetic memory of autophagy

To summarize, (i) exposure to famine, *i.e*. prolonged nutrient starvation, in human is associated to effects on both health span and longevity for the affected individuals as well as their offspring; and (ii) autophagy induction, the cellular response to nutrient starvation, is likewise associated to similar effects on these parameters for the affected organisms. However, the question that remains to be addressed is how autophagy induction could lead to long-term, as well as transgenerational effects? Noteworthy, for an extended period of time, cytoplasmic events were considered of sole importance for this biological process as enucleated cells are still able to show signs of autophagy [57, 93]. However less than a decade ago, this view was been challenged with establishment that the nucleus, with both transcriptomic and epigenetic events, plays central roles in both the regulation and execution of autophagy [94, 95]. Epigenetic modifications refer to both mitotically and meiotically heritable alterations in gene expression that occur without changes in the underlying DNA sequence. Acquired epigenetic modifications, including DNA methylation, histone modifications and non-coding RNAs, can be propagated through mitotic cell divisions. In addition, these non-DNA sequence-based epigenetic information can be inherited across several generations, via transgenerational epigenetic inheritance [96]. A consensus is established that biological factors transmitted from parent to offspring include not only genetic but also epigenetic contribution [97]. Of interest for the current review, in *C. elegans*, as well as in *D. melanogaster*, alterations in several of these epigenetic mechanisms are reported to impact on longevity [96, 98–102]. Hence, an attractive potential mediator of the response to autophagy induction by dietary restriction leading to transgenerational longevity is epigenetics.

**Fig. 3:**
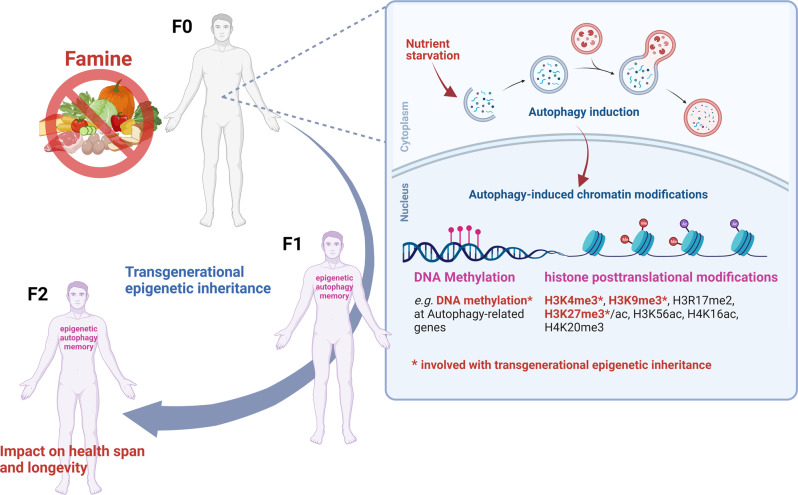
Proposed contribution of autophagy-induced chromatin modifications to transgenerational epigenetic inheritance in response to famine, i.e. nutrient starvation. Exposure of human to famine, *i.e*. nutrient deprivation, is linked to transgenerational effects on health span and longevity in their offspring. Nutrient starvation-induced autophagy is associated to modifications of the chromatin, including DNA methylation and histone posttranslational modifications (including H3K4me3, H3K9me3, H3R17me2, H3K27me3/ac, H3K56ac, H4K16ac and H4K20me3), some of which are reported to contribute to transgenerational epigenetic inheritance. Hence, an epigenetic memory of autophagy could contribute to food deprivation induced effects across successive generations. The image was created with BioRender.com.

Posttranslational modification of core histone proteins, including the acetylation, and methylation of histone tails, is key mechanism of epigenetic regulation. Important groups of histone modifying enzymes in this regulatory network are the histone acetyltransferases (HATs/ KATs for lysine specificity), the histone deacetylases (HDACs), histone methyltransferases (HMTs) and histone lysine demethylases (KDMs). The histone posttranslational modifications can affect the overall chromatin structure and thereby either activate or repress transcription, by modulating the accessibility and binding of transcription factors, and coregulators to the chromatin. The identification of an increasing number of histone marks associated with the regulation and execution of the autophagic process offers an additional attractive conceptual framework to understand the potential long-term response to autophagy (Fig. 3) [94, 103, 104]. Among these autophagy histone marks, histone H4 lysine 16 acetylation (H4K16ac), is found to be reduced upon autophagy induction, through downregulation of the histone acetyltransferase KAT8 (also known hMOF). Indeed, H4K16 deacetylation upon autophagy induction is associated with the downregulation of autophagy-related genes that provides a negative regulatory feedback loop, which in turn serves as a key determinant for survival *versus* death responses [105]. In yeast, H4K16 acetylation regulate cellular lifespan, due to a decrease of the H4K16 deacetylase Sir2 (SIRT1 homolog) expression. Antagonizing the activities of Sir2 or Sas2 (KAT8 homolog), respectively shortened and extended the replicative yeast lifespan through regulation of H4K16ac levels [106, 107]. Duplication of the *sir-2.1* gene, in *C. elegans* extends the lifespan of the worm by 50% [108]. In *D. melanogaster*, Sir2 was found to be directly involved in the calorie-restriction lifespan-extending pathway, as an increase in dSir2 extends lifespan, whereas a decrease in dSir2 blocks the lifespan-extending effect of calorie reduction [109]. From a transgenerational viewpoint, maternally inherited hMOF/KAT8-mediated H4K16 acetylation provides a transcriptomic instruction to the offspring, priming future zygotic gene activation in *D. melanogaster*. The maintenance of H4K16ac from oocytes to fertilized embryos was found to be conserved in mouse [110]. While histone acetylation at lysine residues is generally associated with increased gene expression, methylation of arginine or lysine residues, activate or represses gene expression depending on which residue methylated. Among the different methylated histone marks, two repressive marks, histone H3 lysine 9 trimethylation (H3K9me3), and H3K27me3, and one activating mark, H3K4me3, are reported to be involved in transgenerational epigenetic inheritance in the fruit fly, nematode and mouse [96, 99]. Interestingly, in starved zebrafish myotubes, the transcriptional state of genes involved on the autophagy process (such as *atg4b*, *sqstm1* and *lc3b*) has been shown to be under the strict epigenetic control of all three-histone methylation marks [111]. In mammalian cells, under nutrient rich conditions, the H3K9 methyltransferase EHMT2/G9a binds to the promoter regions of ATG genes including *MAP1LC3* and *WIPI* and represses their expression, whereas during starvation-induced autophagy,, EHMT2/G9a chromatin displacement leads to a reduction in H3K9me3 levels and the transcriptional activation of ATG genes [112]. In human and mouse, the age-associated decrease of the H3K9 histone methyltransferase SUV39H1, which in turn limit the ability of hematopoietic stem cells to generate B lymphocytes, contributes to a decrease in immune function [113]. However, in a progeria (premature aging) mouse model, depletion of SUV39H1 improves DNA repair capacity and extends lifespan of the mice by ~60% [114]. In *C. elegans*, the most commonly used animal model for transgenerational epigenetic inheritance and longevity, worms with increased H3K9me2 levels have a longer lifespan that can be passed down to twenty generations [115]. H3K27me3, another transgenerational heritable repressive methylation mark, is established by the H3K27 methyltransferase EZH2 containing polycomb-repressive complex 2 (PRC2). Interestingly, EZH2 has been shown to be potent regulator of autophagy, where the downregulation of TSC2 by EZH2 elicits MTOR activation, which in turn inhibits autophagy [116]. In the fruit flies, increased E(z)(EZH2 in *D. melanogaster*)-dependent histone H3K27 trimethylation was found to mediate the transgenerational programming on longevity after early-life dietary restriction with shortened lifespan of F0 and F2 offspring [117]. Mutations in *E(z)* or the H3 binding protein *esc*, reduce levels of H3K27me3 and promote longevity in *D. melanogaster* [102]. Likewise in *C. elegans*, downregulation of the H3K27 demethylase UTX-1 increased global levels of H3K27me3 and improved lifespan [118]. Exposure of pregnant rats to environmental toxicants reported to induce epigenetic transgenerational inheritance of phenotype variations or diseases, such as the fungicide vinclozolin or the pesticide DDT (dichlorodiphenyltrichloroethane), resulted in altered H3K27me3 levels at so-called differential methylated histone retention sites that were conserved in sperm of the F3 generation [119, 120]. H3K4me3, the third histone methylation mark associated to transgenerational epigenetic inheritance, decreases with age and has been shown to be linked to regulation of autophagy in yeast and human cells [105, 121]. In nematodes, deficiencies in the H3K4 methyltransferase SET-2 containing ASH-2 trithorax complex, which establish H3K4me3, extend lifespan, while the H3K4 demethylase RBR-2 is required for normal lifespan, supporting that H3K4me3 associated active chromatin is detrimental for longevity [98]. Likewise in the fruit flies, decrease of Lid, RBR-2 orthologue, increase H3K4me3 levels and decrease lifespan [100]. Transgenic mice overexpressing the H3K4 demethylase KDM1A/LSD1, with a resulting decreased in H3K4me2 level in sperm, impairs their offspring health and survival transgenerationnaly [122]. A fourth histone methylation mark, H4K20me3 is localized in constitutive heterochromatin regions and associated with transcriptional repression and is reported to increase with age in rat liver and kidney [123], as well as in human cells derived from patients with Hutchinson-Gilford progeria, a premature aging syndrome [124]. Serum starvation, a prominent inducer of autophagy, has a marked impact on the global level of H4K20me3 in various mouse cell types [125]. In addition, radiation-induced autophagy promotes H4K20 trimethylation on *GABARAPL1* gene leading to autophagy induction in non-small cell lung cancer patients [126]. H4K20me3 is tightly linked to several of the above-mentioned histone marks associated to both the regulation of autophagy and longevity, *e.g*. H4K16ac and H3K9me3. Indeed, H4K20me is deposited through a preceding deacetylation of H4K16ac, and linked to the presence of H3K9me3, thus determining the levels of H4K20me3 throughout the genome [127]. summary, a role for histone posttranslational modifications, *i.e*. histone marks, including those reported to be modulated upon autophagy induction, in mediating epigenetic inheritance is well established in invertebrates but only suggested in the germ cells of vertebrates. DNA methylation is regulated by DNA methyltransferase (DNMTs) enzymes that catalyze the transfer of a methyl group to the fifth carbon of a cytosine ring in cytosine-guanine dinucleotide dinucleotides generating 5-methylcytosine. A role for DNA methylation in the long-term transcriptional control of autophagy was recently uncovered (Fig. 3). In fact, cell exposure even to brief autophagic stimuli, including amino acid starvation, is associated with an upregulation of the *DNMT3A* (DNA methyltransferase 3 alpha) gene expression [128]. The serine/threonine kinase ULK3 (unc-51 like kinase 3)-dependent activation of GLI1 (GLI family zinc finger 1) contributes to the observed transcriptional upregulation of *DNMT3A* gene expression [129]. DNMT3A protein was found to be recruited to and promote the methylation of the promoter region of *MAP1LC3* genes in vitro. Transient autophagy induction was also found to lead to persistent downregulation of *map1lc3* genes expression in zebrafish (*Danio rerio*). In mammals at birth, following the sudden termination of the trans-placental nutrient supply, some organs suffer temporary but severe starvation, which triggers a transient autophagic response [16, 31]. Longitudinal transcriptomic data available for murine lung tissues affected by the early neonatal starvation period revealed that *Map1lc3b* exhibited a significant and sustained decrease in gene expression, whereas *Dnmt3a* gene expression showed over time significant increase in gene expression [128]. A recent demonstration of transgenerational epigenetic inheritance was obtained in methylation-edited mammal, where an engineered epigenetic mutation, *i.e*. DNA methylation of promoter-associated CpG islands, in mice was shown to be inherited across four generations of offspring. The acquired CpG islands methylation was subjected to demetylation in parental primordial germ cells, the gamete precursors, but the heritable epigenetic memory was found to be subsequently re-established in the next generation at the post-implantation epiblast E6.5 embryonic stage [130]. Hence, these recent observations provide a concrete step toward demonstrating DNA methylation based transgenerational epigenetic inheritance in mammals, which may have implications in our understanding of the long-term and heritable effects of autophagy induction.

Looking at the human population-based historical cohort studies referred above, it appears that DNA methylation-mediated transgenerational epigenetic inheritance also occurs in human upon exposure to famine. Indeed, in the population-based cohort study from Suihua China, blood DNA methylomes performed on 138 subjects across two generations revealed 961 and 503 differentially methylated sites, in the F1 and F2 generations respectively, between the famine and non-famine exposed groups. Remarkably, 19 differentially methylated sites, including in the loci of *CUX1*, *PPARGC1A*, *ELMO1* and *AGTR1*, that have been linked to autophagy regulation, were shown to be conserved across generations suggesting that DNA methylation modifications occurring after nutrient restriction can be subject to transgenerational transmission [16]. It should be noted that whereas conservation of DNA methylation profiles across generations has been reported in human cohorts exposed to hunger, as well as in human cohort of long-lived individuals such as nonagenarian and centenarian, conservation of histone marks reported in animal model to contribute to the regulation of autophagy as well as transgenerational epigenetic inheritance remain to be demonstrated.

## Conclusion and perspective

Overall, the discovery that autophagy induction is associated to epigenetic alterations, including DNA methylation and histone posttranslational modifications, and that some of this epigenetic information can be inherited by future generations, offer a conceptual frame for the understanding of the long-term effect of food deprivation including in the context of human famine (Fig. 3). Additional epigenetic modifications induced by nutrient starvation and autophagy induction, beyond the above-described ones, will certainly emerge as further epigenetic regulator of health span and lifespan. For example, in *C. elegans*, small RNAs induced gene silencing has been shown to be able to persist over several generations via transgenerational inheritance [131]. In fact, a set of starvation-induced small RNAs are transmitted transgenerationally, providing a mean for starved worms to control the expression of relevant genes in consecutive generations [132]. Evidence from human famines, as well as animal studies indicates that nutrient starvation affect the health and lifespan of the famished individuals as well as their progeny. However, these studies also indicate that these effects depend on (1) the sex of both the individuals exposed to nutrient deprivations as well as the sex of the offspring and (2) the time of hunger exposure, prenatal (in utero) versus postnatal exposure to nutrient deprivations. The exact mechanisms behind these observed differences in the response to starvation and associated transgenerational epigenetic inheritance remain to be established. However, it should be noted that sex-dependent differences in adaptation to famine have long been appreciated [133, 134]. Autophagy shows sex-dependent differences in both physiological and pathological processes, with the potential impact of sex steroid hormones and a role for the X chromosome (reviewed in [135, 136]). Even constitutive autophagy exhibits a sex bias, as autophagy levels investigated in spinal cord and skeletal muscle tissues of wild-type and unchallenged mice at 40–120 days of age was shown to exhibit significant sex and tissue specific differences [137].

Moreover, sex can be influenced by epigenetic changes promoted by the environment that can make one sex more adaptable to a new environment than the other, and thus promote its survival [138]. The first studies on environmental sex determination (ESD) linked elevated temperature levels led to an increase of males in European sea bass due to an increase of DNA methylation levels at the promoter of the enzyme responsible for estrogen synthesis, cyp19a1a [139]. Half-smooth tongue sole (*Cynoglossus semilaevis*), a marine fish that has both chromosomal genetic sex determinant and temperature-dependent ESD, shows DNA methylation as the responsible mechanisms for the transition from GSD to ESD. Studies in *C. semilaevis* demonstrate that ESD it is not limited to cyp19a1a gene rather across genes involved on the sex determination network, with an enrichment of differential DNA methylation patterns. Moreover, transgenerational epigenetic sex inheritance studies performed in that model shows that offspring from a normal male (ZZ) and a female (ZW) exposed to 28 °C during development induced genetic females (ZW) into pseudomales. Pseudomales was subsequently crossed with one normal female to produce F1 pseudomales and females. Interestingly, offspring of pseudomales can spontaneously develop into a functional pseudo-male without the environmental factor involved, due to novel DNA methylation patterns during the juvenile fish at high temperature alters the developmental fate of the gonadal cells. These findings suggest that these new environmental-dependent DNA methylation patterns are imprinted in the genome and transferred to the offspring [140]. In mice, Jmjd1a, the histone methylase responsible for H3K9me2 mark controls the expression of the mammalian Y chromosome sex-determining gene (SRY). Interestingly, XY mice with non-mutated *Sry* gene have developed as female which suggest the role of epigenetics modifications in mammalian sex determination [141].

Regarding the dramatic difference in observed effects of the transgenerational epigenetic inheritance of the response to nutrient starvation exposure on both health span and longevity in human depending on whether there was a fetal or prepubertal childhood exposure, developmental epigenetic events can be brought forward as possible explanation. Indeed, the fetal period in human, as well as numerous organisms, is a period associated to robust epigenetic (re-)programming events that contribute to proper development. In fact, prenatal exposure to environmental factors, beyond nutrient deprivation, such as stress, infections, toxins, can disrupt gene expression programming in the fetus, resulting in long-term epigenome alterations and developmental deficits that can affect the individual later in life [142, 143]. One can speculate that the effects observed in in utero exposed individuals, as well as their offspring, is the result of the sum of autophagy-induced epigenetic modifications and perturbation of developmental epigenetic programs. In contrast, the long-term effects of postnatal hunger could be the sole or predominant result of autophagy-induced transgenerational epigenetic inheritance. Interestingly, detailed analysis of the effect on lifespan extension of targeting various autophagy-related genes in *C. elegans* using RNA interference revealed that their manipulations had contrasting detrimental or beneficial effects on worm longevity depending on whether the gene silencing was performed maternally or adult. These results suggest that autophagy induction may not always be beneficial to longevity but may also function to restrict lifespan in the nematode [58]. In summary, one can envision that autophagy associated epigenetic changes in DNA methylation, RNA silencing and histones posttranslational modifications are passed down from one generation to the next, affecting the epigenetic age and lifespan not only of the exposed but also subsequent generations. As a possible illustration of this concept in human, as previously highlighted nonagenarians and centenarians have been shown to exhibit both differentially expressed autophagy genes, as well as unique heritable DNA methylation profiles across generations which could contribute to longevity [43, 44].

## Supplementary information

checklist
